# Minimally invasive treatment for glioblastoma through endoscopic surgery including tumor embolization when necessary: a technical note

**DOI:** 10.3389/fneur.2023.1170045

**Published:** 2023-04-21

**Authors:** Tomohiro Sakata, Motoki Tanikawa, Hiroshi Yamada, Ryota Fujinami, Yusuke Nishikawa, Shigeki Yamada, Mitsuhito Mase

**Affiliations:** Department of Neurosurgery, Nagoya City University Graduate School of Medical Sciences, Nagoya, Japan

**Keywords:** endoscope, glioblastoma, tumor embolization, cylinder surgery, key-hole surgery

## Abstract

**Background:**

Although there have been some reports on endoscopic glioblastoma surgery, the indication has been limited to deep-seated lesions, and the difficulty of hemostasis has been a concern. In that light, we attempted to establish an endoscopic procedure for excision of glioblastoma which could be applied even to hypervascular or superficial lesions, in combination with pre-operative endovascular tumor embolization.

**Methods:**

Medical records of six consecutive glioblastoma patients who received exclusive endoscopic removal between September and November 2020 were analyzed. Preoperative tumor embolization was performed in cases with marked tumor stain and proper feeder arteries having an abnormal shape, for instance, tortuous or dilated, without passing through branches to the normal brain. Endoscopic tumor removal through a key-hole craniotomy was performed by using an inside-out excision for a deep-seated lesion, with the addition of an outside-in extirpation for a shallow portion when needed.

**Results:**

Endoscopic removal was successfully performed in all six cases. Before resection, endovascular tumor embolization was performed in four cases with no resulting complications, including ischemia or brain swelling. Gross total resection was achieved in three cases, and near total resection in the other three cases. Intraoperative blood loss exceeded 1,000 ml in only one case, whose tumor showed a prominent tumor stain but no proper feeder artery for embolization. In all patients, a smooth transition to adjuvant therapy was possible with no surgical site infection.

**Conclusion:**

Endoscopic removal for glioblastoma was considered to be a promising procedure with minimal invasiveness and a favorable impact on prognosis.

## Introduction

Glioblastoma, one of the most common primary brain neoplasms, comprises 15% of all intracranial neoplasms and 60–75% of astrocytic tumors (1). Its treatment outcomes have improved only incrementally over the last several decades, with a tragic course, having a mean survival time fewer than 2 years after diagnosis, despite many efforts having been made in various fields, including surgical resection, radiation therapy, chemotherapy, and treatments based on novel concepts (2–4). Regarding surgical treatment, maximizing the extent of resection, which must be balanced finely with minimizing morbidities, has been confirmed to be essential for extending patients’ survival time (1, 5, 6). A broad variety of refinements to achieve optimal resection have been made through the utilization of multiple modalities, including preoperative techniques such as diffusion tensor imaging (7), functional magnetic resonance imaging (MRI) (8–10), or magnetoencephalography (11), and intraoperative modalities such as image guidance (12), photodynamic diagnosis, for instance using 5-aminolevulinic acid (13), functional mapping *via* awake surgery, intraoperative ultrasound imaging, or intraoperative MRI (14, 15). Nevertheless, the prognosis of this devastating disease has not been substantially improved. Given this state of affairs, anything that can contribute to ameliorating the bleak prognosis of this disease is to be greatly desired.

Current neurosurgical procedure is undergoing what could be considered a transitional era, with a conventional operating microscope (OM), an endoscope, or an exoscope employed divergently according to the lesion or to surgeons’ preferences. The OM, which appeared in the 1960s, revolutionized improvement in neurosurgical outcomes (16, 17) by providing a stable, illuminated, magnified, 3-dimensional view, though at the cost of sacrificing the surgeon’s ergonomic comfort insofar as a bulky object between the operative field and the surgeon’s eyes (18). In contrast, an exoscope, developed through advances in digital imaging, offers an operative view equivalent to an OM and superior ergonomics, though even experts in microsurgery require a certain amount of practice to attain a level clinically comparable to maneuvering under an OM (19). Since an exoscope, as with OM, utilizes illumination from the outside, a sufficiently large opening is necessitated; nevertheless, blind spots are still likely to occur in the deep areas (18). On the other hand, an endoscopic procedure with superior ergonomics can be performed through a narrow opening and corridor and reduce blind spots in deep areas (20). However, its maneuverability is limited by space, and in comparison to other devices, some time is required to acquire a sufficient level of skill. In addition, the current technology of endoscope provides inferior images of the operative field compared to other devices.

**Figure 1:**
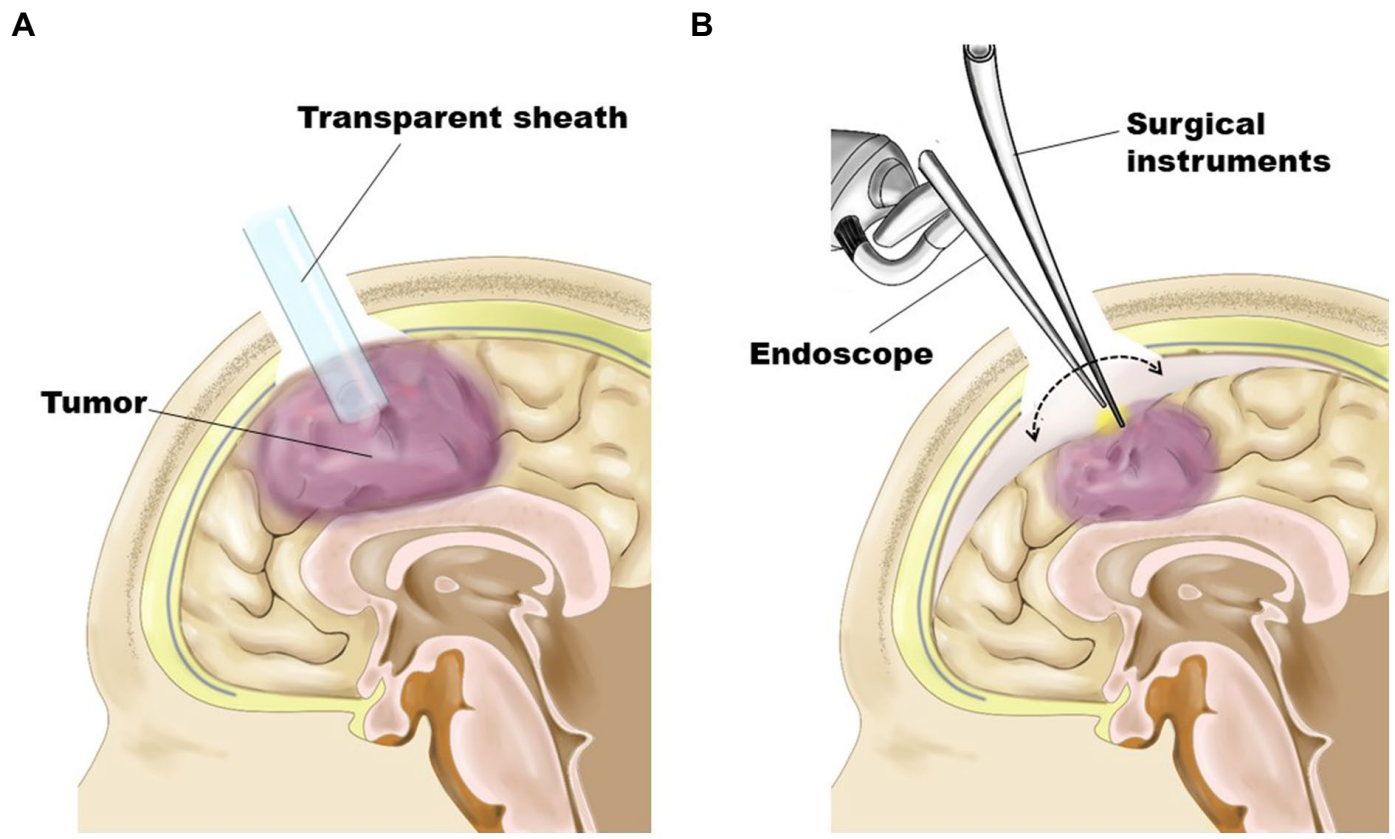
Schematic representation demonstrating surgical procedures. **(A)** As the first step, the tumor was removed in an inside-out fashion through a transparent sheath inserted into the tumor’s center under the guidance of a neuronavigation system. **(B)** For tumors having a shallow portion, the residual part was removed in an outside-in fashion after obtaining enough space by subsidence of the brain surface through substantial tumor volume reduction.

Introducing endoscopic surgery through a small opening for glioblastoma removal might benefit patients by virtue of limiting the invasiveness of the procedure, reducing the burden on patients, simplifying wound healing, decreasing the chance of infection, and contributing to the smooth transition to adjuvant treatment. In fact, some reports have described the utilization of an endoscope for glioblastoma surgery through a narrow transparent sheath, and it has been confirmed that endoscopic procedures could be performed as efficaciously as with an OM (21, 22). However, the same reports recommended that the procedure be essentially limited to deep-seated lesions. Moreover, the procedure necessitates initially entering the middle of a lesion, which can sometimes be vascular-rich, creating difficulty in achieving hemostasis through a limited corridor. Therefore, in this study, we attempted to develop an optimal and minimally invasive surgery for glioblastoma, which could be applied even to hypervascular or superficial lesions, by utilizing endovascular tumor embolization and endoscopic removal through an outside-in procedure following inside-out decompression (Figure 1).

## Methods

All procedures performed in this study were in accordance with the 1964 Declaration of Helsinki and its later amendments and were reviewed and approved by the Institutional Review Board (IRB) at Nagoya City University (IRB number: 60-20-0187). Additionally, written informed consent was obtained from all the participants or appropriate surrogate decision-makers. Between September and November 2020, 6 consecutive glioblastoma patients, who were set to undergo surgical resection, were enrolled in this study. Data regarding clinical manifestation, neuroimaging, intraoperative videos, and surgical outcomes were analyzed.

## Surgical procedure

### Endovascular tumor embolization

**Figure 2:**
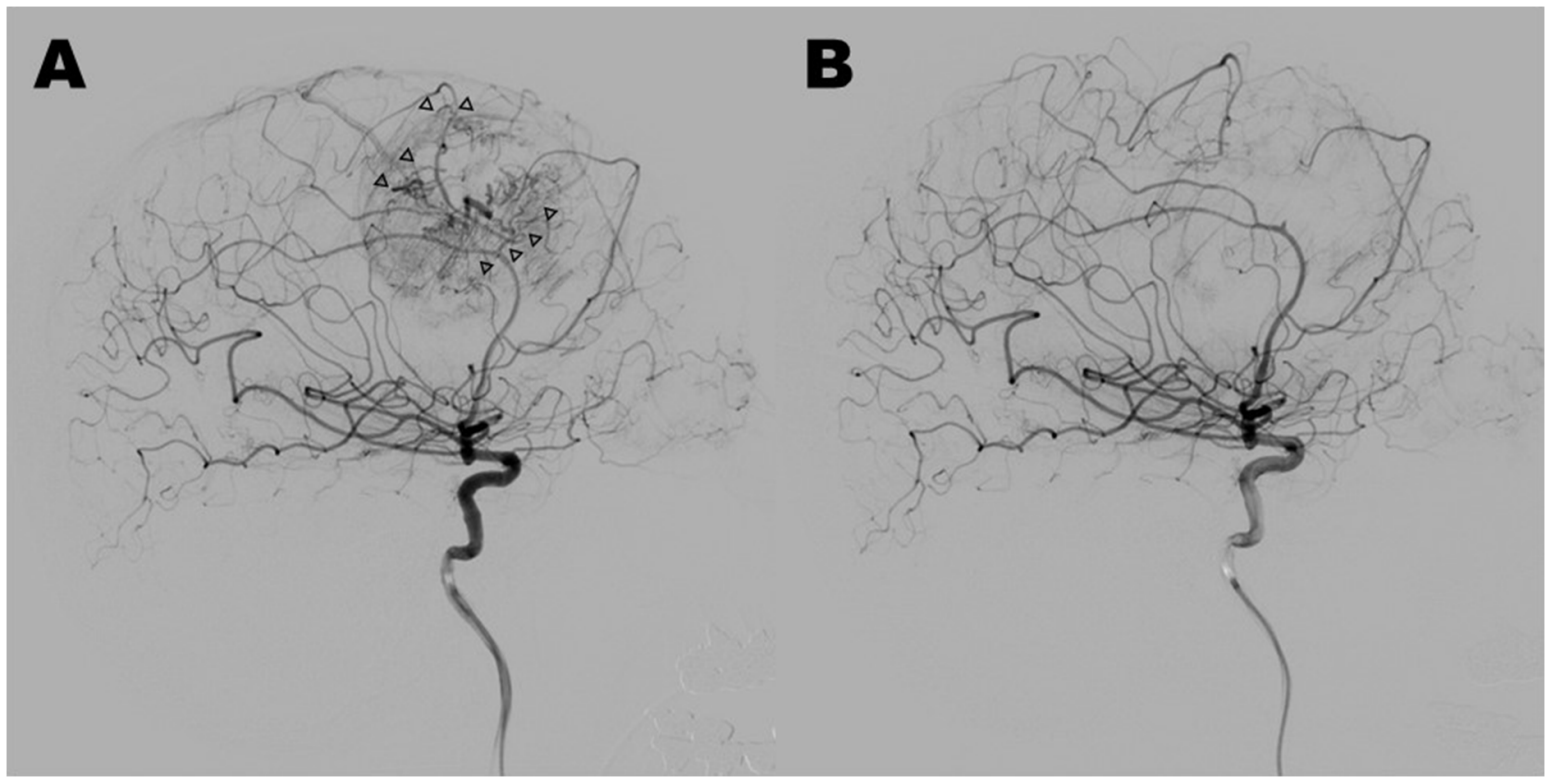
Case 4: lateral view of pre-embolization **(A)** and post-embolization **(B)** in digital subtraction angiograms of the right internal carotid artery. Marked tumor stain and prominent feeding arteries with abnormal shapes (arrowheads) disappeared through tumor embolization with 20% NBCA, and normal arteries were preserved.

Tumor embolization was performed if there was a marked tumor stain and prominent feeding arteries with abnormal shapes, for instance, tortuous or dilated, without passing through branches to the normal brain (Figure 2A). A cone-beam computed tomography (CT) was effective for confirming these findings. Under general anesthesia, a 5Fr guiding catheter was inserted into the main trunk, and a flow-guide microcatheter (Marathon; ev3 Neurovascular, Irvine, CA, United States) was advanced into the feeder vessel as close to the tumor as possible. The tumor was embolized with approximately 20% NBCA (Figure 2B). Platinum coils were also used for feeder vessel occlusion to prevent migration of NBCA. After this procedure, tumor removal proceeded continuously, or if time was required until tumor excision, general anesthesia was maintained in the intensive care unit to avoid brain swelling.

### Endoscopic tumor removal

**Figure 3:**
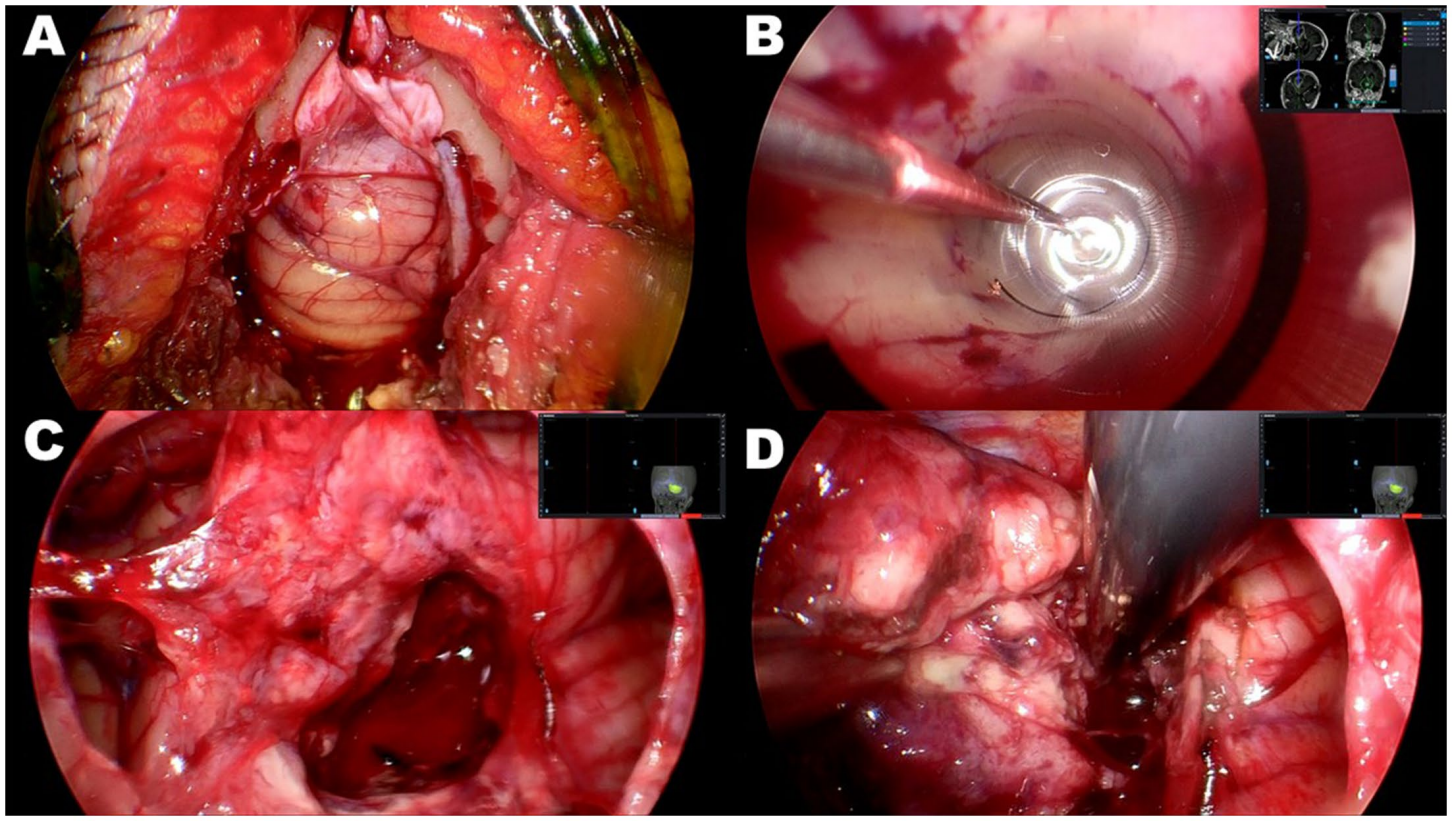
Case 2: intraoperative photographs of right cerebellar glioblastoma. **(A)** Key-hole craniotomy approximately 20 mm in diameter. **(B)** Insertion of 10 mm transparent sheath into center of tumor through guidance of neuronavigation system. The tumor was subsequently removed in an inside-out fashion. **(C)** After obtaining sufficient space by subsidence of brain surface through substantial tumor volume reduction, transparent sheath was removed. **(D)** Residual portion of tumor resected in an outside-in fashion through sufficient cortical incision.

Surgical procedures of endoscopic tumor removal were performed with the aid of 4-mm rigid endoscopes with 0 or 30-degree angled lenses fixed with an exclusive holder (HD-EndoArm, Olympus, Tokyo, Japan), a high-speed drill, an ultrasonic surgical aspirator, and a series of endoscopic surgical instruments with a slender or single shaft. Under general anesthesia, patients were placed with their head positioned so that the location of a key-hole craniotomy was highest in the operative field and the axis from there to the center of the tumor was slightly tilted forward, allowing both hands of the surgeon to assume an ergonomically advantageous position. A key-hole craniotomy, which was simulated preoperatively to determine the proper size and location using an image workstation, was set precisely and performed using an image guidance system (SealthStation S8; Medtronic, Minneapolis, MN, United States) (Figure 3A). The lateral edge of the craniotomy was conically widened to allow, to the extent possible, manipulation over a wide range (Figure 1). After insertion of a transparent sheath with a diameter of 10 mm (NeuroPort; Olympus, Tokyo, Japan) into the center of the tumor, guided by the image guidance system (Figure 3B), an endoscope was introduced. Initially, internal decompression of the tumor was conducted using suction or an ultrasonic surgical aspirator in an inside-out fashion while attaining hemostasis rigorously through cauterization by bipolar forceps with a slender or single shaft, or hemostatic material such as fibrin glue. If a lesion had only a deep-seated portion, it could be entirely removed through repetition of these procedures. For a lesion that also had shallow portions, after obtaining substantial subsidence of the brain surface and sufficient working space by internal decompression, the transparent sheath was taken out (Figure 3C), and the remaining part was resected in an outside-in fashion through a sufficient cortical incision (Figure 3D). Finally, after confirming hemostasis, BCNU wafers were placed over the extent of the tumor bed. The highlights of the procedure can be seen in Supplementary Video S1.

## Results

**Figure 4:**
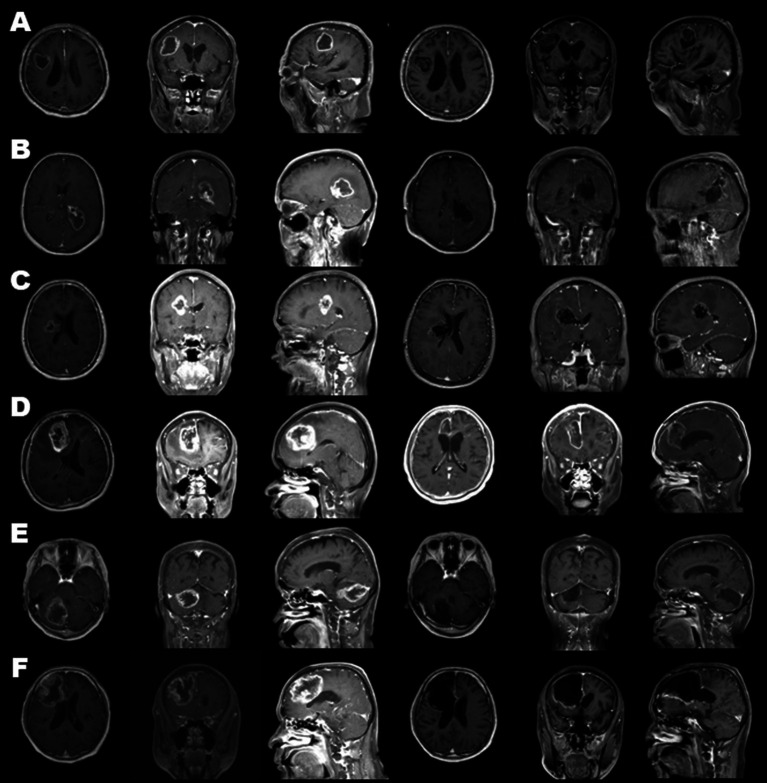
Preoperative (three columns on the left) and postoperative (three columns on the right) gadolinium-enhanced magnetic resonance images of all cases. Cases 1, 2, 3 **(A–C)** were treated through tumor removal only in an inside-out fashion. Cases 4, 5, 6 **(D–F)** combined excision in an outside-in fashion. As a result, gross-total resection in cases 1, 4, and 5 **(A,D,E)** and near-total resection in cases 2, 3, and 6 **(B,C,F)** was achieved.

Endoscopic removal was successfully performed in six glioblastoma cases as an initial treatment through a key-hole craniotomy approximately 20 mm in diameter. Clinical features and surgical outcomes of six cases were shown in Table 1. Prior to resection, endovascular tumor embolization was performed in four cases, in which marked tumor stain and proper feeding arteries were detected by preoperative angiography. In all four cases, there were no complications related to tumor embolization such as ischemia or brain swelling. In three cases, where tumor developed only in a deep-seated area, the portion identified on gadolinium-enhanced T1-weighted MRI was removed entirely in one and near-totally in two cases, in an inside-out fashion (Figures 4A–C). In the remaining three cases, the procedure was supplemented with outside-in excision, and gross-total resection in two patients (Figures 4D,E) and near-total resection in one patient was achieved (Figure 4F). In two patients where a premotor area was involved, tumor removal could be performed with minimal deterioration of hemiparesis (one grade on MMT) by identifying and preserving the motor strip and pathway through motor-evoked potential mapping, after creating space by initially removing the forward portion of the lesion, a maneuver which was considered to be relatively safe. One other patient had transient upper extremity motor weakness postoperatively. Intraoperative blood loss exceeded 1,000 ml in only one case, where the patient did not receive preoperative tumor embolization due to the lack of a proper feeder for embolization, despite having a prominent tumor stain. In all patients, a smooth transition to adjuvant therapy was possible with no surgical site infection.

**Table 1:** Clinical characteristics and outcomes of the six patients with fully endoscopic glioblastoma removal.

Case	Age, Sex	Size (mm)	Location	Preoperative embolization	Endoscopic procedure	Extent of resection	Blood loss	Pathological diagnosis	Survival time	Complications
1	70 years, M	32 × 31 × 30	Rt frontal lobe	No	I-O	GTR	336 ml	Glioblastoma, IDH-wildtype	123 weeks, alive	Upper limb motor weakness (transient)
2	59 years, F	40 × 34 × 29	Lt parietal lobe	No	I-O	NTR	1,048 ml	Glioblastoma, IDH-wildtype	49 weeks	None
3	72 years, F	28 × 24 × 36	Rt frontal lobe	Yes	I-O	NTR	106 ml	Glioblastoma, IDH-wildtype	29 weeks	Deterioration of hemiparesis (persistent)
4	68 years, F	53 × 51 × 36	Rt frontal lobe	Yes	O-I following I-O	GTR	580 ml	Glioblastoma, IDH-wildtype	129 weeks, alive	None
5	73 years, F	55 × 31 × 42	Rt cerebellum	Yes	O-I following I-O	GTR	354 ml	Glioblastoma, IDH-wildtype	45 weeks	None
6	66 years, M	69 × 53 × 49	Rt frontal lobe	Yes	O-I following I-O	NTR	651 ml	Glioblastoma, IDH-wildtype	45 weeks	Deterioration of hemiparesis (persistent)

I-O, inside-out style; O-I, outside-in style; GTR, gross total resection; NTR, near total resection.

## Discussion

This study successfully demonstrated the feasibility and effectiveness of an endoscopic excision for glioblastoma not only for the deep-seated portion *via* the inside-out procedure through a narrow transparent sheath, but in addition even for the shallow portion *via* the outside-in procedure. Since the goals of surgical resection of glioblastoma are to provide ample tissue for an accurate histological and genomic diagnosis and continued study of the disease, to relieve symptoms, and to extend patient survival by achieving maximal cytoreduction without morbidity, it was thought that this procedure could realize these aims to nearly the same extent as the ordinary microscopic procedure. Predicted difficulty in manipulation capability through narrow opening and corridor produced virtually no stress even during the inside-out phase through a 10 mm transparent sheath. Furthermore, in the subsequent process preceded by thorough internal decompression, the space through the key-hole craniotomy could be sufficient for adequate removal *via* the outside-in procedure.

Bleeding is one of the most likely complications during the removal of glioblastoma because glioblastoma is often highly vascularized by neoformed vessels, as microvascular proliferation is one of its diagnostic hallmarks (23). Therefore, endovascular tumor embolization was considered indispensable for the smooth and safe progression of an endoscopic glioblastoma excision procedure if the target lesion was vascular-rich. In fact, blood loss relatively increased in the case of hypervascular lesion, which could not be embolized due to a lack of a suitable feeder artery. In such cases, thorough preparation of hemostatic devices and transfusion blood will be required. On the other hand, because glioblastoma is an intraparenchymal tumor, proper selection of intratumoral vessels to be embolized, which were characteristically tortuous and dilated in shape (24), and which ended in the tumor without passing through branches to the normal brain, was considered very important. A cone-beam CT, which does not require particularly exceptional equipment (25), was useful for identifying those findings in this study. In either case, endovascular tumor embolization has not been standard treatment for glioblastoma, and even in this study, it was only performed in four cases. Therefore, a future study including a more significant number of cases will be necessary to establish the efficacy and safety of this technique.

Surgical intervention is confirmed to cause a neurophysiological reflex response involving hypothalamic–pituitary–adrenal axis activation, and results in complex neuroendocrine, inflammatory, metabolic cascade, and immunological responses (26–29). Experimentally, inflammatory mediators, such as CINC1, IL-8, TNF-α, and NO, have also been found to increase proportionately depending on the length of the skin incision experimentally (29). In addition, the reduction of the inflammatory response has been proven with the laparoscopic procedure compared to open surgery (30–32). Therefore, surgical intervention, including skin incision, muscle dissection, and craniotomy, should be minimized not just for the sake of reducing pain and better cosmetic results but also from the standpoint of mitigating the surgical stress response, and the endoscopic procedure was considered to be suitable for its realization even in surgery of glioblastoma.

Surgical site infections (SSI) after cranial surgery, which are an inevitable complication and have been reported to occur in 1–16% of patients (33–42), especially in glioblastoma surgery, would not only lead to the discontinuation of postoperative treatment but would also affect patient survival (33). While various risk factors for SSI have been assumed, e.g., number of operations (34–38, 41), duration of operation (35, 37, 39, 40), emergency operation (37), cerebrospinal fluid leakage (34, 35, 37, 39, 42), CSF drainage (35, 39), and American Society of Anesthesiologists score (>2) including body mass index and diabetes mellitus (34–36, 38, 42), there have also been reports that craniotomy itself could constitute a risk factor (40, 41). Therefore, by using endoscopic resection for glioblastoma through key-hole craniotomy as in this study, it may be possible to reduce SSI to a level similar to that of other organs (43, 44), and facilitate a smooth transition to postoperative therapy.

The largeness of a lesion would not be a contraindicating factor for this procedure since, in principle, the larger the lesion, the larger the space that can hypothetically be secured through an internal decompression. On the other hand, it can be inherently difficult in this procedure, which employs endoscopic surgery through a narrow opening, to remove a lesion which extends widely in a shallow area. Even for such lesions, however, successful removal could be achieved in this study by making space through internal decompression, provided there was the appropriate volume and depth. This study was not able to establish the relationship between the extent of the surface and volume in the deep portion, where this procedure can be successfully utilized. A lesion close to eloquent areas, for which the use of brain mapping would be essential for removal, could be assumed to be another limitation of this procedure. Brain mapping may also be possible if a lesion involving the eloquent area has a significant amount of lesion in the adjacent non-eloquent area, as was the situation in two cases in this study, whose premotor lesions were successfully resected by using motor evoked potential mapping. Moreover, this procedure, performed through small skin incision and craniotomy, may be suitable for awake surgery because of the reduced burden on the patient. Notwithstanding, since the present study is only a preliminary report, including a small number of patients and only a few types of lesions, further studies with more cases are needed to clarify the efficacy and safety of this endoscopic procedure for glioblastoma and to eliminate such limitations.

## Conclusion

Endoscopic removal of glioblastoma using an inside-out style excision for a deep-seated lesion was considered to be a promising procedure, with the addition of an outside-in style extirpation for a shallow portion if needed. This procedure could be performed *via* small skin incision and key-hole craniotomy less invasively and had a positive effect on patients’ prognosis, facilitating a smooth transition to postoperative adjuvant therapy. In addition, endovascular tumor embolization, when it was needed before resection, was also considered beneficial. However, a more extensive number of patients will need to be evaluated in future studies to confirm the efficacy and safety of this procedure.

## Data availability statement

The raw data supporting the conclusions of this article will be made available by the authors, without undue reservation.

## Ethics statement

The studies involving human participants were reviewed and approved by Institutional Review Board at Nagoya City University. The patients/participants provided their written informed consent to participate in this study. Written informed consent was obtained from the individual(s) for the publication of any potentially identifiable images or data included in this article.

## Author contributions

TS: conceptualization, project administration, acquisition, methodology, investigation, resources, data curation, formal analysis, and writing–original draft. MT: methodology, investigation, resources, and writing–review and editing. HY, RF, YN, and SY: methodology, investigation, and resources. MM: conceptualization, funding acquisition, and supervision. All authors contributed to the article and approved the submitted version.

## Conflict of interest

The authors declare that the research was conducted in the absence of any commercial or financial relationships that could be construed as a potential conflict of interest.

## Publisher’s note

All claims expressed in this article are solely those of the authors and do not necessarily represent those of their affiliated organizations, or those of the publisher, the editors and the reviewers. Any product that may be evaluated in this article, or claim that may be made by its manufacturer, is not guaranteed or endorsed by the publisher.
